# How patients with insomnia interpret and respond to the consensus sleep diary: a cognitive interview study

**DOI:** 10.1186/s41687-024-00695-y

**Published:** 2024-02-20

**Authors:** Christina Bini, Carina Hjelm, Amanda Hellström, Kristofer Årestedt, Anders Broström, Christina Sandlund

**Affiliations:** 1 Department of Neurobiology, Care Sciences and Society, Division of Family Medicine and Primary Care, Karolinska Institutet, Alfred Nobels allé 23, Huddinge, SE-141 83 Sweden; 2 Academic Primary Health Care Centre, Region Stockholm, Solnavägen 1E, Stockholm, 113 65 Sweden; 3 Department of Health, Medicine and Care, Nursing and Reproductive Health, Linköping University, Linköping, 581 83 Sweden; 4 Faculty of Health and Life Sciences, Linnaeus University, Universitetskajen 1, Kalmar, SE-392 31 Sweden; 5 Department of Research, Region Kalmar, Lasarettsvägen 8, Kalmar, SE-39185 Sweden; 6 Department of Nursing, School of Health and Welfare, Jönköping University, Jönköping, 551 11 Sweden; 7 Department of Clinical Neurophysiology, Linköping University Hospital, Linköping, 581 85 Sweden; 8 Department of Health and Caring Sciences, Western Norway University of Applied Sciences, Bergen, Vestlandet 5020 Norway

**Keywords:** Sleep wake disorder, Sleep diary, Validation, Cognitive interviewing, Patient reported outcome measures, Response bias

## Abstract

**Objective/Background:**

The Consensus Sleep Diary (CSD) is widely used to assess subjective sleep. Psychometric evaluations and focus-groups support its validity and clinical usefulness, but further research into its validity is needed. The aim of the study was to evaluate a Swedish translation of the CSD regarding test content and response processes in patients with insomnia.

**Patients/Methods:**

In connection with translating the CSD into Swedish, we used cognitive interviewing to evaluate test content and the response process, that is, how people make decisions when responding to survey items. Cognitive interviews were conducted with 13 primary health care patients with insomnia disorder (mean age, 49 years; SD 15.5). Iterative, reparative analysis was used to investigate test content. Descriptive deductive analysis was used to investigate interview transcripts for the themes of the cognitive model: comprehension, retrieval, decision process, and judgement. Together, the themes explain the response process when responding to a patient-reported outcome measure.

**Results:**

The overall comprehension of the CSD could be affected by poor adherence to the instructions (comprehension). Patients had difficulty with recall if they did not complete the diary immediately in the morning and just before bedtime (retrieval). They could have problems deciding how to respond to certain items because they imbued sleep-related concepts with extra meaning (decision process), and had trouble finding response alternatives nuanced enough to describe their experience of sleep and tiredness (judgement).

**Conclusions:**

This study contributes knowledge on how the instrument is perceived and used by care-seeking patients with insomnia. In this context, the CSD exhibits known flaws such as memory lapses if the diary is not filled in directly in the morning. To increase the accuracy of patients’ responses, therapists should support patients in reading the instructions.

## Background

Insomnia is a common sleep disorder characterized by persistent problems to initiating sleep and/or maintaining sleep, as well as significant daytime impairments regarding work, school, or social life [2]. The cognitive model of insomnia [18] suggests that hyperarousal plays an important role in maintaining insomnia. Alongside biological factors and stressful life events, the hyperarousal is influenced by misconceptions about sleep and subsequent worry, resulting in selective monitoring and heightened attention to physical and psychological needs, counterproductive safety behaviors, increased worry, physiological arousal, and emotional distress [20].

According to international guidelines, patient reported sleep diaries are important tools for assessing and treating insomnia [28, 32]. They are used to gather subjective information on sleep and sleep habits that is not addressed by objective measures such as actigraphy. Sleep diaries are administered in both paper and electronic form [36], for at least seven consecutive days [7], and the information typically gathered includes bedtime, sleep onset latency, number and duration of nocturnal awakenings, wake-up time, the time the person got out of bed, and the frequency and duration of daytime napping [10, 30]. They might also gather information on sleep quality and how rested the person felt on awakening [22], as well as on factors such as hypnotic drug use and lifestyle habits that can impact sleep (e.g., alcohol and caffeine consumption, food intake, and exercise) [30].

In both research and clinical practice, it is important to assess sleep with a standardized, validated sleep diary. Thus, in 2005, a panel of sleep experts undertook a multi-step process to create such an instrument, the Consensus Sleep Diary (CSD) [11]. They tested its content in focus groups of good sleepers, people with insomnia, and people with sleep apnea. All CSD items have undergone Lexile analysis [11], a quantitative method of measuring text complexity to enhance reading comprehension [34]. Further, the items have been psychometrically evaluated by comparing responses with actigraphy results and Insomnia Severity Index (ISI) scores [27]. Previous studies on the clinical usefulness of the CSD have shown that the diary items are easy to understand, not too complex, and measure what they are intended to measure [12, 24]. The CSD is recommended to be used in the clinical assessment of insomnia [32]. The CSD is available in two versions: a core version, consisting of nine questions on night related aspects of sleep, and an expanded version, consisting of 20 questions including questions on daytime related aspects of sleep. Both versions include general instructions and specific instructions for each question. The questions are answered using various response formats, including free-text responses (e.g., bedtime and details of hypnotics), yes or no responses, number of occurrences (e.g., number of awakenings), and subjective ratings on a Likert scale (e.g., sleep quality). Additionally, there is a field for own comments (e.g., I have a cold). A detailed description of the CSD instructions, items and matrix is presented elsewhere [11].

Ideally, evaluations of how well an instrument does what it was intended to do, such as how well the CSD measures subjective sleep, should be based on evidence about test content (which includes item themes, wording, and format) and internal structure (indicating whether components align with the intended construct), psychometric properties, the consequences of testing, and the response process (how people make decisions when responding to survey items) [1]. To the best of our knowledge, test content and patients’ response process when filling in the CSD remain uninvestigated.

Evaluations of these aspects are important to ensure that target respondents in each new context interpret the text as intended and can respond truthfully to the items [1]. Therefore, the aim of the study was to evaluate a Swedish translation of the CSD regarding test content and the response processes in patients with insomnia.

## Methods

### Design

This qualitative evaluation study of the CSD was conducted in connection with translating and adapting the CSD for use in Swedish research and clinical contexts. We used the method of cognitive interviewing (CI) described by Willis [42, 43] to evaluate the CSD. The method allowed us to take two approaches: first, a *reparative (finding and fixing)* approach to evaluate test content of the Swedish translation [25], and second, a *descriptive* approach to analyze the response process. The descriptive approach enabled us to investigate the themes of comprehension, retrieval, decision process, and judgement, predefined themes in the cognitive model of survey response [37].

CI is one of the most used and validated methods for examining test content of patient-reported outcome measures (PROMs) such as the CSD, as well as for investigating the response process. It can also be used to develop and test the item validity of PROMs intended for use in clinical assessment and treatment [25]. The study followed the Cognitive Interviewing Reporting Framework [5].

### Translation and pre-testing of the consensus sleep diary

The research group translated the English version of the expanded CSD (also referred to as CSD-M) [11] into Swedish with permission from Carney, who developed the diary. The translation was guided by the principles of good practice for translating and culturally adapting patient-reported outcome measures [40] and the principles of CI [26, 42].

Two independent translators first forward translated the CSD into Swedish through cognitive debriefing with the members of the research group, all native speakers of Swedish. That version was thereafter independently back translated into English by two native speakers of English. The researchers then compared the Swedish version and both English back translations with the original diary to harmonize the language [11].

The preliminary Swedish version of the CSD was evaluated in fifteen healthy volunteers (women and men, 18–75 years) recruited from two communities in southern Sweden. One round of cognitive retrospective probing interviews was conducted after the volunteers had used the sleep diary for one week. The answers from the fifteen volunteers were anonymized, placed in a matrix, summarized, and then analyzed using the reparative approach. At this point, the researchers agreed on small verbal changes to the Swedish translation of the CSD based on the analyses. The developer was contacted for approval in connection with every change made in the translation. The preliminary version was then evaluated regarding test content and response process in patients with insomnia.

### Evaluation of test content and response process

#### Participants and setting

Participants were recruited in 2021 at a primary health care center in Stockholm, Sweden. Patients who sought care for insomnia symptoms were routinely scheduled for individual assessment by a district nurse trained in assessing insomnia. The district nurse used purposive sampling [29], inviting patients to participate in the study until she had gathered a varied group representative of the heterogeneity of patients with insomnia (e.g., sex, age, education level, comorbidities, medications, and insomnia severity).

To be eligible for inclusion, patients had to meet the Diagnostic and Statistical Manual of Mental Disorders (DSM-5) [2] criteria for insomnia and be ≥18 years. Patients were excluded if they had a severe psychiatric disorder (i.e., psychotic disorder, bipolar disorder, severe depression) or cognitive disorder (i.e., dementia), worked night shifts, or could not speak or read Swedish. Sixteen patients met the study criteria and were invited to participate. After receiving oral and written information about the study, all provided written informed consent to take part. All patients received instructions on how to use the diary (e.g., “If you forget to complete the diary or are unable to finish it, leave the diary blank for that day”) and were advised to read the instructions before and alongside the keeping of the diary. For unknown reasons, three dropped out before the interviews, so the study included a total of 13 patients (Table 1).

**Table 1:** Demographic characteristics of the participants (n = 13)

Variables	
Age, mean (SD)	49 (15.5)
**Sex, n (%)**	
Female	8 (61.5)
Male	5 (38.5)
**Employment status, n (%)**	
Employed	6 (46.2)
Student	2 (15.4)
Retired	5 (38.5)
**Education level, n (%)**	
University	7 (54.8)
High school	6 (46.2)
**Marital status, n (%)**	
Cohabiting partner	7 (54.8)
Living alone	6 (46.2)
**Country of birth, n (%)**	
Sweden	12 (92.3)
Other	1 (7.7)
**Current psychiatric disorders, n (%)**	9 (69)
Anxiety	7 (54.8)
Depression	5 (38.5)
OCD	1 (7.7)
**Current somatic disease, n (%)**	4 (30.7)
Chronic pain	2 (15.4)
Hormonal disorder	1 (7.7)
Cardiovascular disease	1 (7.7)
**ISI score, mean (SD)**	16.5 (4.5)
**Current treatment for insomnia, n (%)**	13 (100)
Pharmacological^a^	7 (54.8)
Non-pharmacological^b^	4 (30.7)

*Abbreviations ISI* Insomnia Severity Index, *n* number, *OCD* obsessive-compulsive disorder

^a^Pharmacological treatment included sedative drugs, anti-anxiety medication, medication for depression, and melatonin

^b^Non-pharmacological treatment included psychological and/or behavioral treatment, as well as sleep hygiene and general health recommendations

#### Developing the semi-structured interview guide

The Question Appraisal System (QAS) [42, 43], a checklist for ensuring that the interview covers the test content and the themes in the cognitive model, was used to develop a semi-structured interview guide (Appendix). The questions in the guide were influenced by the responses from the interviews conducted to pre-test the Swedish translation. The sections of the interview guide covered the steps in QAS, and the guide consisted of probing statements and questions, such as “Tell me about your week with the diary,” and “What does ‘nap’ mean to you?” It also included probing questions, such as “Tell me more” and “Can you expand on that?” The questions and interview technique were tested in the research group before the interviews [43].

#### Cognitive interviews

**Fig. 1:**
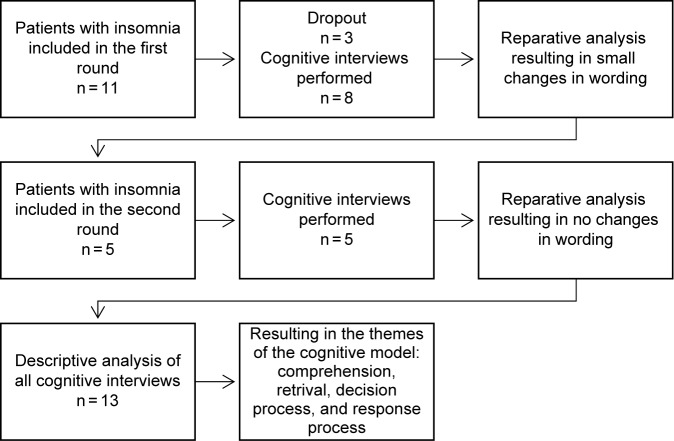
Overview of the cognitive interview process

An overview of the CI process is shown in Fig. 1, which included 13 patients in total. The first eight patients were consecutively provided with the preliminary Swedish version of the CSD and asked to use it daily for two consecutive weeks. One week after each patient started to fill in the diary, the interviewer called to schedule an interview. The interviewer then carried out the first round of cognitive interviews during the second week of diary use. After the first round of interviews, no new information was found. Two of the authors collaborated to revise and modify the diary in keeping with the findings.

Five additional patients with insomnia were then provided with the revised CSD. The interviewer carried out a second round of interviews using the same methods as in the first round. No new actionable findings emerged after the second round, and the authors judged that the Swedish version of the CSD had reached final form.

Of the in total 13 interviews, 10 were conducted as videoconferences by the digital platform Teams, two by telephone, and one face-to-face. All interviews were audio recorded and lasted for a median of 23 min. The recordings were pseudonymized and transcribed verbatim. Notes were taken after each interview.

#### Analytical process

The analysis of the cognitive interviews had two objectives: to improve items and achieve a better understanding of the test content and patients’ response process when filling in the CSD [25]. These objectives were reached via two analytical approaches, the reparative approach and the descriptive approach.

##### Reparative approach

The reparative approach was deductive, top down, and iterative [25, 43]. We used the strategy of question feature coding (Model 3) [43]. The headings of each section in the interview guide were used as themes in the analysis. The themes included *reading, instructions, clarity, assumptions, memory/knowledge, sensitivity or bias, response categories*, and *other problems*.

First round, reparative approach: After each interview in the first round and before transcription, the interviews were summarized to retain the overall impression of the interview and improve reflexivity in the analytical process. After transcription, a matrix that summarized each participant’s answer to every question was created. Quotations illustrating the summary were also placed in the matrix. Each summary was labeled with a code, and the codes were sorted into categories. Two authors analyzed whether the categories should lead to changes in the translation or the design of the CSD. Second round, reparative approach: It comprised five interviews, and followed the same analytical process as the first round.

##### Descriptive approach

The interviews from the first and second round (n = 13) were included in the descriptive analysis. Like the reparative approach, the descriptive [25] was deductive and top-down [43]. We once again used the strategy of question feature coding [43], this time to analyze the transcripts for the four themes in the cognitive model: *comprehension, retrieval, decision process*, and *judgement* [37, 41]. *Comprehension* explained what the patients believed the CSD was about and what they believed specific terms in the diary meant. *Retrieval* explained how patients recalled relevant information. It included both the kind of information necessary to prompt recall and the recall strategies used. The third theme, *decision process,* covered two areas: “motivation” and “sensitivity/social desirability.” The first area was about the efforts made to respond to the question as correctly as possible. The second, sensitivity/social desirability, was about whether the participant responded truthfully to questions even when they found it socially undesirable to do so. The last theme, *judgement,* was about whether the CSD response alternatives provided patients with the opportunity to respond in a way that matched their internally generated responses to the questions. The authors analyzed each summary in the matrices and sorted them into the cognitive model themes.

## Results

### Test content

The reparative analysis involved determining whether the categories called for changes in the translation or design of the CSD, with changes made only if they maintained the diary’s fidelity. After the second round of interviews, the authors concluded that further adjustments would fundamentally change the CSD. Patient feedback on the Consensus sleep diary (CSD) that did not lead to any modifications are shown in Table 2.

**Table 2:** Patients comments on the CSD that did not result in any modifications

Suggested changes	Decision based on analysis
Remove either question 6a and 6b or question 7, as they yield the same answer.	Declined because of too significant change to the original
Raise the rating scale by two levels for questions 9 and 10 on the 5-point scale.	Declined because of too significant change to the original
Place the alcohol-related question after the coffee-related question.	Declined because of too significant change to the original
Move question 14 about hypnotics to the daytime section.	Declined because of too significant change to the original
Allow patients to use smartwatches for tracking sleep patterns.	Smartwatches vary in reliability and do not provide insights into the subjective sleep experience, which is the primary focus of sleep diaries
Include a question regarding sugar intake.	Declined because of too significant change to the original
Add a question about nighttime coping strategies when sleep is disrupted.	Declined because of too significant change to the original
Ensure there is a comments section in the daytime section.	Declined because of too significant change to the original
Include a question addressing sleep quality.	Declined because of too significant change to the original
Include a question regarding the effects of hypnotics.	Declined because of too significant change to the original

### Response process

#### Comprehension

Patients experienced the CSD easier to comprehend, but more extensive than other sleep diaries they had previously used. They appreciated the presence of a comment field for “other issues” but thought it could be even larger. One explained, “You may have a lot of other problems that affect sleep, and then it will not fit in the field” (patient [P] 3).

Patients thought questions 6a (“What time was your final awakening?”), 6b (“After your final awakening, how long did you spend in bed trying to sleep?”), and 7 (“What time did you get out of bed for the day?”) were confusing because the responses to 6a and 6b provided the same information as question 7. Thus, patients thought they had not understood the questions correctly, and the instructions did not always alleviate their confusion.

Patients found some wording and phrases in the CSD problematic. Did “time to fall asleep” start when you turned off the light or when you closed your eyes? What counted as a “nap”? Only deliberate sleep during the day, or also dozing off, for example, when watching TV? Furthermore, patients overestimated the size of a “glass of alcohol,” even though the amount in a standard glass was explained in the instructions. Patients said that they could find it hard to judge their sleep quality with the rating scale. Despite instructions on how to think about sleep quality, patients could still be confused. They were not certain whether to base their assessment on the hours they slept, how rested they felt when they woke up, or the amount of deep sleep they had. One participant said, “Is [the amount of] deep sleep the quality of sleep? Can you judge sleep quality when you’re unconscious?” (P13).

Patients pointed out that the CSD was designed to be answered by people who had an organized, well-structured life. For example, patients who lacked a predetermined wake-up schedule, such as those who were retired, unemployed, or had irregular sleep habits had problems answering question 6c (“Did you wake up earlier than planned?”).

#### Retrieval

Patients could find it hard to establish the routine of completing the CSD twice a day. One participant suggested that notifications from an app could help them remember to complete the diary. If patients did not fill in the diary soon after waking up or in the evening before going to bed, recall became difficult, and they said they had to guess their answers. Those living with a partner could use their partner to help them remember and sometimes even asked the partner to complete the diary for them.

Patients employed recall strategies that aligned with their personal interpretation of sleep quality (question 11). Patients whose beliefs regarding sleep quality deviated from the provided definition, or those who failed to read or comprehend the instructions, might, for example, use a smartwatch to gauge sleep quality based on the duration of uninterrupted sleep or the amount of deep sleep recorded on the watch’s hypnogram. A general comment regarding the CSD was that it should be accessible in the form of an app since using a paper diary was considered outdated: “It feels somewhat analog to note all this data; it could have been done more swiftly and easily” (P7).

#### Decision process

Tiredness and stress affected motivation. When tired or stressed, patients did not have the energy to read or reread the instructions, even when they felt unsure. Instead of turning to the instructions, some repeatedly re-read the question to ensure they had understood the meaning correctly.

Motivation was further affected by the insight the CSD provided. For instance, patients could learn that they slept more than they realized, which could be calming and encouraging. One patient said: “I have slept a bit better when I’ve actually written down when I sleep, when I wake up, and how long I stayed in bed. So, I have had a positive impression of this, actually” (P10). On the other hand, the urge to complete the diary correctly could negatively affect sleep, which reduced motivation.

Although some found it easy to quantify the time they spent asleep and the time they spent in bed in hours and minutes: “Now, I am very mathematically inclined, so I had no issues with that” (P12), others found the effort challenging: “Yeah, well. It, yeah, it was a bit tricky, actually. I have to admit that. I don’t know how to count, you have to sit like this and count on your fingers” (P1). Patients described a feeling of not answering the questions seriously if they responded subjectively. They could perceive that the need to correctly quantify time was at odds with not watching the clock while in bed. Some patients created systems to keep track of their nocturnal awakenings, for example by making notes on a piece of paper every time they woke up. However, these systems were not effective because they could not understand their own notes in the morning. “One night I had written something really strange in the middle of the night which I did not understand anything of in the morning. It was not a good strategy” (P11).

Regarding questions 12a and 12b, which were about alcohol consumption, patients stated that others might not answer truthfully, but they themselves would, since they had nothing to hide. Patients thought that admitting that they used sleep medication at all, took too high a dose, or took it too frequently (question 14) would negatively impact the therapists’ view of them.

#### Judgement

It could be hard for patients to find a response alternative, or a field in the CSD that matched their experience. For example, they could not find an alternative for the state between sleeping and being awake, which some described as “half sleep.” Patient number 3 said, “If you’re half-sleeping, then it’s really hard to answer, because I don’t know if I’ve been asleep or not. When did I actually wake up? I don’t know!” Patients specifically noted that the mismatch between CSD alternatives and their subjective experience of half sleep could make question 4 about the number of nocturnal awakenings and question 6b about early awakening hard to answer.

Patients thought the two 5-point graded scales, one about sleep quality and the other about how rested they felt, did not have enough choices to cover their experiences of these variables. One patient explained, “It’s too small of a spectrum in sleep quality and the other one with grades” (P4).

Finally, the question about how much sleep medication the patient had taken was problematic because it was to be filled in before going to bed. Patients usually took their medication in the middle of the night when they had given up on sleep. They would therefore have preferred to respond to this question in the morning. “It’s strange that I’m supposed to note the time when I take a sleeping pill in the middle of the night. I take them during the night and not in the evening,” said patient number 11.

## Discussion

This study evaluated a Swedish translation of the CSD regarding test content and patients’ response process. The results illuminate how patients with insomnia understand and interpret the CSD items, strategies they use to remember their answers, how they decide what to answer, and whether the CSD has response alternatives that match their intended answers.

Patients with insomnia appreciated the CSD instructions since they missed guiding instructions in previous sleep diaries they had used. However, most patients encountered some difficulties in understanding the instructions. One possible reason could be that symptoms of insomnia include cognitive impairments, such as disorientation [16] and reduced ability to concentrate [31]. However, it could also be due to the previously known limitations of sleep diaries, regardless of whether the patient has a sleep diagnosis or not [12]. For example, people often tend to overestimate their sleep onset, and time awake after sleep onset. Additionally, memory lapses can occur when the diary is not filled out immediately upon waking [11]. That’s partly why Carney et al. [11], took measures to create the written instructions.

To overcome the problems with memory lapses, measuring instruments like smartwatches was suggested to facilitate data recording. However, commercial devices have their limitations, showing significant variations based on factors such as brand, gender, age, and concurrent illnesses [3]. Most importantly, these devices do not capture a person’s experiences of sleep, which is crucial for diagnosing insomnia and assessing its severity [32]. Even some actigraphy that are specially calibrated to measure sleep, and that are scientifically validated have been shown to be unable to distinguish sleep diary variables, except total sleep time, between patients with insomnia and normal sleepers [33]. This also renders them ineffective as a sole assessment tool, hence, it is worthwhile to continue investigating possibilities and developing a more effective instrument for measuring subjective sleep. As suggested in the present study, a potential solution to reduce memory lapse problems could involve an electronic diary that reminds patients to complete both in the morning and evening. For example, the Clinical Outcome Assessment (eCOA) system, which is designed to enhance consistency and compliance in clinical trials [15]. According to a previous study, there are no significant differences in sleep parameters between paper and electronic sleep diary [36].

We found that the patients’ difficulties with recall led to strategies for filling in the CSD that resulted in problematic (counterproductive to patients) and likely incorrect responses. It can be hard to create new habits for completing questionnaires and logbooks [9]. In the current study, as in previous studies [11, 12], patients had trouble remembering to complete the CSD and trouble with recall when responding after a delay. They therefore used strategies that could lead to biased responses, such as guessing or asking their partners. Since the assessment and treatment of insomnia rely on subjectively self-perceived reported sleep, it becomes problematic if it is fabricated or filled in by relatives. It could also be possible that patients took these actions to please the therapist, a known patient strategy that clinicians should be aware of when using PROMs [8, 17]. Regardless of why patients employed problematic strategies for completing the CSD, the resulting inaccuracies could negatively affect treatment because the form and content of cognitive and behavioral therapy for insomnia (the recommended treatment) is based on information from the diary [13, 28, 32]. Therapists should therefore consider reminding patients to leave responses blank if they cannot remember the answer.

Sleep-related hyperarousal [18] may play an important role in several of our findings. For example, the patients desired more nuanced response alternatives than what the diary offered, such as additional options on the sleep quality scale. People tend to focus on the basic need they lack, such as food, shelter, or sleep in the case of people with insomnia [14]. It is thus natural that people with insomnia pay unusually close attention to sleep which could lead them to distinguish details of the experience that people without insomnia generally do not attend to.

Another example comes from our findings about the judgement theme. Patients experienced something they described as “half sleep” and subsequently had difficulty responding to several items in the diary because “half sleep” was not a response alternative. The experience of “half sleep” might correspond to non-REM stage 1 and/or stage 2 sleep as measured by polysomnography [39]. If awakening occurs at this stage, a person will not perceive themselves as having been asleep [6]. It is possible that sleep-related hyperarousal makes patients with insomnia more aware of theses stages than normal sleepers [19, 38].

### Methodological considerations

To the best of our knowledge, this evaluation study was the first to use CI to investigate the test content of the Swedish translation of the CSD and the response process of patients with insomnia when using the diary. Evidence from different sources improves the validity of an instrument [1]. The evidence from this study therefore complements the results of previous validation studies.

Throughout the translation process, the researchers adhered to international guidelines and communicated regularly with the developer of the CSD. These two steps enhance the study’s validity, ensuring that the results can be applied to the original version. This is the reason why we refrained from making substantial revisions during the repetitive analyses, which would have led to a translation that deviates from the original, jeopardizing the transferability of the results.

There is no standard approach to CI, so the method is carried out in a variety of ways [25]. However, there are standards for how CI research should be reported [5], which the present study followed. An additional strength of the study was the careful planning and clear structure of the interview guide, constructed in accordance with the QAS system, which increases the credibility of the findings by ensuring that the interviews covered all four cognitive phases of a response process [43].

During analysis, the authors reflected on and discussed how their different backgrounds affected their perspectives and interpretations. The ongoing discussions in the research group brought diverse points of view to the analysis and ensured that no single perspective was dominant. The method was described in detail to facilitate the reader’s ability to judge transferability [21, 23], and quotes were used to support the confirmability of the findings [4].

In CI, the goal of sampling is to gather varying perspectives rather than to achieve a representative sample [4, 42]. Participants were of different ages and sexes, had comorbidities, and had different treatments for insomnia (Table 1). Therefore, the number of participants was considered sufficient to investigate test content and response process [35]. The heterogeny of the study sample may make the findings transferable to patients with insomnia treated in a variety of clinical settings. They may not be transferable to patients with other sleep disorders, such as sleep apnea and circadian rhythm disorders. Therefore, we suggest that future studies should evaluate the response process of other target populations that use the diary. Moreover, the present study evaluated the expanded version of the CSD. Patients’ response process may be different when using the shorter core version.

## Conclusions

This study added knowledge about the validity of the CSD by investigating a fourth aspect of the validity testing framework, the response process. The study illuminates issues with the CSD regarding test content that can be considered if the original version is modified in the future. Moreover, it contributes with evidence on how the diary is perceived and used by care-seeking patients with a diagnosis of insomnia. In this context, the CSD exhibits known flaws, which could be partly related to the characteristics of the disorder when insomnia is recognized for its impact on decision-making, action-taking, comprehension, and judgement. The findings stress the importance of researchers and clinicians’ awareness of the potential for response bias when they interpret findings based on CSD data in patients with insomnia.

## Data Availability

The datasets used and analyzed during the current study are available from the corresponding author on reasonable request.

## Appendix

### Interview guide—consensus sleep diary

#### Cognitive interviews with patients about their experience and comprehension of completing consensus sleep diary

Open question

- Tell me briefly about your weeks with the diary and the instructions?

Stage 1 (Comprehension)

- Tell me how it was to read and understand the questions. – Tell me more!
- Did you have to read repeatedly? – Tell me more!
- Were there any questions you had to think extra about? Tell me more?

Stage 2 (Instructions)

- Tell me about your experience with the instructions. -Could you expand on that?
- Were there any instructions you had to read repeatedly?
- Why? Tell me more!
- Could you give example on how it could be improved?

Stage 3 (Clarity)

- Could you describe the meaning of these words and phrases to me? Nap, Time to fall asleep, a glass of alcohol, and waking up earlier than planned? (Based on the pilot testing of the diary) Tell me more!

- Were there other words that you think could be missed comprehended? If so, how would you phrase it?
- Are there any items, in general, that could be missed comprehended? Tell me more!

Stage 4 (Assumptions)

- Were there any questions you found strange answering? If so, tell me more!
- And if so, how would you phrase the question?

Stage 5 (Memory/knowledge)

- How was recalling the questions? Tell me more! Why? How would you suggest an improvement?

- How was counting the sleep hours? Tell me more!
- How did you feel, what did you think?

Stage 6 (sensitivity/bias)

- Was something sensitive to answer? Why? Tell me more. How would you have asked?

- Is there some wording you would like to change? Why?

Steg 7 (Response categories)

- How was the order of the questions? Tell me more.
- Were you able to find correct response categories that matched your intended answer? What did you miss?

Steg 8 (Other)

- Were there any other problems with the diary or the instructions that I haven’t asked about? Tell me more!

Final question

- If you have discussed the diary with your next of kin, what have you talked about? Tell me more.

## Abbreviations

CI: Cognitive Interviewing
CSD: Consensus Sleep Diary
ISI: Insomnia Severity Index
PROM: Patient reported outcome measures
QAS: Question Appraisal System
